# Ventricular Tachycardia in Repaired Double Chambered Right Ventricle - Identification of the Substrate and Successful Ablation

**DOI:** 10.1016/s0972-6292(16)30462-4

**Published:** 2012-01-31

**Authors:** Raja J Selvaraj, Pakkirisamy Gobu, Thulaseedharan S Ashida, Geofi George, Jayaraman Balachander

**Affiliations:** Department of Cardiology, Jawaharlal Institute of Postgraduate Medical Education and Research

**Keywords:** ventricular tachycardia, ablation, double chambered right ventricle

## Abstract

A 35 year old female presented with recurrent ventricular tachycardia 5 years after she had undergone surgical repair of double chambered right ventricle. Electroanatomical mapping showed a localised scar in the apex with double potentials and good pace map. Ablation here resulted in non-inducibility of ventricular tachycardia. We hypothesise that the scarring in the apex is the result of sustained pressure overload and becomes arrhythmogenic similar to the apical scar in patients with mid-ventricular hypertrophic cardiomyopathy.

## Case

A 35 year old female presented elsewhere with a rapid, broad complex tachycardia and presyncope. She was cardioverted and started on intravenous amiodarone, but developed recurrent tachycardia over the next two days. Sustained ventricular tachycardia of the same morphology was easily inducible with ventricular burst pacing during an electrophysiology study and she was referred to our institute for ablation or implantable cardioverter defibrillator implantation.

Review of her past medical history showed that she was diagnosed with double chambered right ventricle (DCRV) five years back with severe obstruction in the mid right ventricle, tricuspid regurgitation (TR) and dilated right atrium. She underwent surgical repair in the form of resection of the obstructing muscle bundles in the right ventricle (RV) and tricuspid annuloplasty. After the surgery, she had class II NYHA dyspnea with paroxysmal atrial fibrillation. Echocardiogram at our institute showed preserved left and right ventricular function, severe tricuspid regurgitation, dilated right atrium and no residual gradient in the right ventricle. ECG recorded during the tachycardia showed a broad QRS tachycardia at 230 bpm with an LBBB morphology and left axis deviation, deep S waves in V5-6 and atrioventricular dissociation.

**Figure 1:**
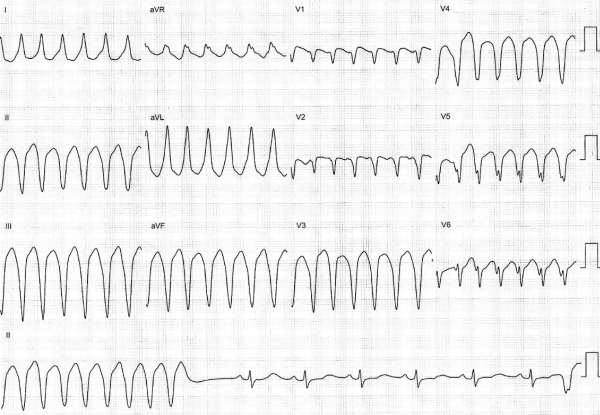
12 lead recording of VT induced during the electrophysiology study. The VT morpholohy is identical to the clinical VT.

**Figure 2:**
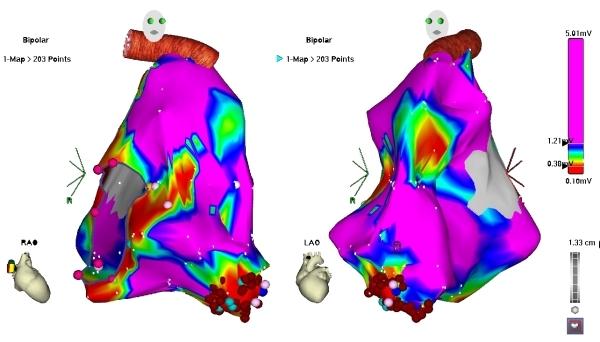
RAO and LAO views of the voltage map of the RV. Scarring and distortion of geometry is seen in the mid RV. A discrete scar is seen in the RV apex.

**Figure 3:**
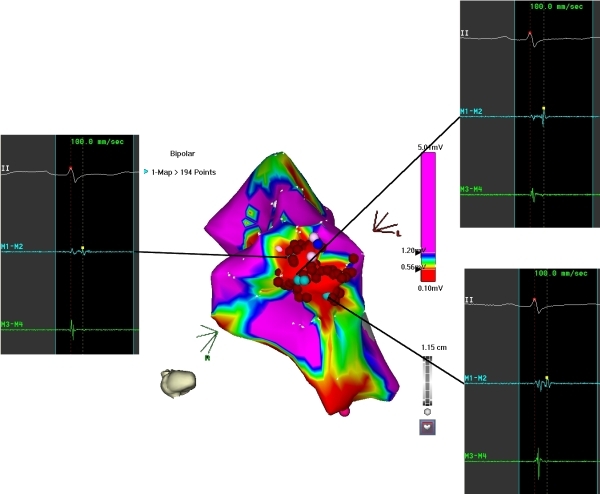
Voltage map of the RV rotated to show the scar in the apex. Representative signals from three regions within the scar show double potentials with the second component occurring well beyond the end of the QRS.

**Figure 4:**
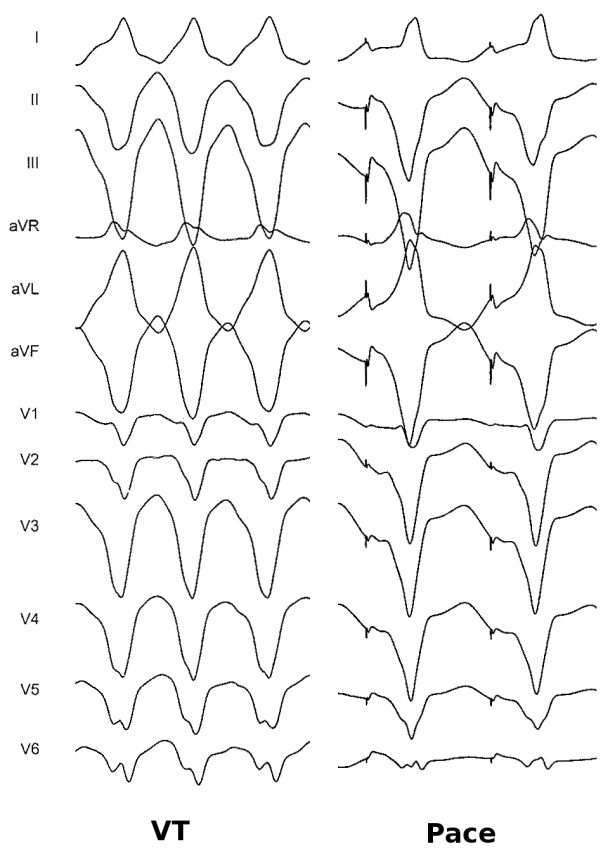
Pace mapping from the anterior border of the scar in a region with delayed potentials. Recorded VT is shown on the left and the paced QRS morphology is on the right. Note the latency from the pacing stimulus and the 11/12 match.

Ablation was planned with the aid of electroanatomical mapping (CARTO XP, Biosense Webster). Programmed stimulation was performed from a quadripolar catheter in the RV apex. Ventricular tachycardia (VT) at a cycle length of 350 ms, identical to the clinically documented tachycardia, was consistently induced with ventricular burst pacing, but always terminated spontaneously after 10-20 beats (Figure 1). Programmed stimulation with upto two extrastimuli did not induce any sustained tachycardia. A large curve, irrigated, 4 mm tip, deflectable ablation catheter (Navistar, Biosense Webster) was used to map the RV in sinus rhythm. There was difficulty positioning and maneuvering the catheter because of tricuspid regurgitation and dilated right atrium, so a long deflectable sheath (Agilis NxT, St. Jude) was used to stabilise the catheter and improve reach. Voltage map revealed scarring in the mid RV at the site of muscle bundle resection, but there were no late potentials and pace mapping here showed QRS morphology very different from the clinical tachycardia. Distortion of the geometry in the mid RV was evident. Further mapping showed an area of focal scarring in the RV apex (Figure 2). Double potentials with a late second component could be recorded from the scar. Pace mapping from the anterior edge of this scar showed the closest pace map to the clinical VT (Figure 3). Since no sustained VT could be induced and the non-sustained VT did not last long enough for entrainment maneuvers, substrate based ablation was performed by targeting areas with late potentials within the apical scar (Figure 4). A total of 16 RF applications were delivered at 30W and flow rate of 25 ml/min. Post ablation, burst pacing and programmed stimulation from the RV with upto 3 extrastimuli failed to induce VT. At follow up 2 months after the procedure, the patient has had no recurrences.

## Discussion

Double chambered right ventricle (DCRV) is a rare congenital cardiac anomaly characterised by obstruction in the RV dividing it into a proximal high pressure chamber and distal low pressure chamber. Treatment is by resection of the obstructing muscle band during childhood or youth. Rare patients have been described who have reached adulthood without surgical repair. Ventricular tachycardia has sometimes been reported in these patients [1,2]. In these reports, the VT is uniformly of LBBB morphology with leftward or inferior axis and late precordial transition. The pathogenesis of VT is commonly assumed to be secondary to structural anomalies related to the hypertrophied muscle band, ventriculotomy scar or the endocardial scar at the site of muscle resection. In the only report on mapping and ablation, the VT was mapped to the left ventricle in a patient who had concomitant aortic valve replacement [3].

In our patient, we found an area of scarring at the RV apex remote from the mid right ventricle where muscle resection was performed. Good match on pace mapping from this region, the presence of late potentials during sinus rhythm and non-inducibility of tachycardia after ablation support the importance of this scar in arrhythmogenesis in this patient. It is well recognised that perfect pace map may not be obtained from the isthmus in cases of scar related reentry because of myocardial capture by the antidromic wavefront and or presence of functional block during VT that is absent in sinus rhythm [4]. Magnetic resonance imaging with late gadolinium enhancement to delineate scar could have proved useful in identifying the substrate before the procedure in this patient.

We hypothesise that the mid ventricular obstruction, with the resultant high pressure in the apex, results in ischemia and fibrosis. This would be analogous to the development of apical scarring and aneurysms with venticular tachycardia in patients with hypertrophic cardiomyopathy and mid ventricular obstruction [5]. This hypothesis is compatible with the appearance of ventricular tachycardia only in patients unrepaired into adulthood and frequent resolution with relief of obstruction. In our patient, the fibrosis and scarring was possibly advanced enough to result in ventricular tachycardia despite relief of the obstruction. By targeting the areas with diastolic activation, corresponding to channels of viable tissue in the scar with delayed conduction, we were able to make the VT non-inducible.

## Conclusion

VT in patients with DCRV repaired in adulthood is an uncommon but well described problem. We report the first such case where electroanatomical mapping has enabled characterisation of the substrate. We found the origin of VT to be in an apical scar and hypothesise that this is analogous to the apical scar in patients with HCM and mid-ventricular obstruction.

## References

[R1] Alvarez M (2006). Sustained monomorphic ventricular tachycardia associated with unrepaired double-chambered right ventricle. Europace.

[R2] Matsuo S (2007). Cardioverter defibrillator implantation in a patient with double chambered right ventricle. The International Journal of Cardiovascular Imaging.

[R3] Kim J (2008). Electrical Storm Late after Surgery for a Double-Chambered Right Ventricle, Aortic Regurgitation and a Ventricular Septal Defect: A Case of Successful Catheter Ablation. Korean Circ J.

[R4] Brunckhorst CB (2004). Identification of the Ventricular Tachycardia Isthmus After Infarction by Pace Mapping. Circulation.

[R5] Sanghvi NK (2007). Sustained ventricular tachycardia in apical hypertrophic cardiomyopathy, midcavitary obstruction, and apical aneurysm. PACE.

